# A novel uterine leiomyoma subtype exhibits NRF2 activation and mutations in genes associated with neddylation of the Cullin 3-RING E3 ligase

**DOI:** 10.1038/s41389-022-00425-3

**Published:** 2022-09-07

**Authors:** Miika Mehine, Terhi Ahvenainen, Sara Khamaiseh, Jouni Härkönen, Siiri Reinikka, Tuomas Heikkinen, Anna Äyräväinen, Päivi Pakarinen, Päivi Härkki, Annukka Pasanen, Anna-Liisa Levonen, Ralf Bützow, Pia Vahteristo

**Affiliations:** 1 Applied Tumor Genomics Research Program, University of Helsinki, Helsinki, Finland; 2 Department of Medical and Clinical Genetics, University of Helsinki, Helsinki, Finland; 3 iCAN Digital Precision Cancer Medicine Flagship, Helsinki, Finland; 4 A. I. Virtanen Institute for Molecular Sciences, University of Eastern Finland, Kuopio, Finland; 5 Department of Obstetrics and Gynecology, Helsinki University Hospital, Helsinki, Finland; 6 Department of Pathology, University of Helsinki and HUSLAB, Helsinki University Hospital, Helsinki, Finland

**Keywords:** Cancer genomics, Gynaecological cancer

## Abstract

Uterine leiomyomas, or fibroids, are the most common tumors in women of reproductive age. Uterine leiomyomas can be classified into at least three main molecular subtypes according to mutations affecting *MED12*, *HMGA2*, or *FH*. FH-deficient leiomyomas are characterized by activation of the NRF2 pathway, including upregulation of the NRF2 target gene *AKR1B10*. Here, we have identified a novel leiomyoma subtype showing AKR1B10 expression but no alterations in *FH* or other known driver genes. Whole-exome and whole-genome sequencing revealed biallelic mutations in key genes involved in neddylation of the Cullin 3-RING E3 ligase, including *UBE2M*, *NEDD8*, *CUL3*, and *NAE1*. 3′RNA sequencing confirmed a distinct molecular subtype with activation of the NRF2 pathway. Most tumors displayed cellular histopathology, perivascular hypercellularity, and characteristics typically seen in FH-deficient leiomyomas. These results suggest a novel leiomyoma subtype that is characterized by distinct morphological features, genetic alterations disrupting neddylation of the Cullin 3-RING E3 ligase, and oncogenic NRF2 activation. They also present defective neddylation as a novel mechanism leading to aberrant NRF2 signaling. Molecular characterization of uterine leiomyomas provides novel opportunities for targeted treatment options.

## Introduction

Uterine leiomyomas, or fibroids, are benign tumors that arise from the smooth muscle wall of the uterus. They are the most common neoplasms affecting women during reproductive years. Although non-cancerous, leiomyomas frequently cause a variety of symptoms including pressure upon adjacent organs, abnormal uterine bleeding, pelvic pain, and impaired fertility [1]. Leiomyomas are the leading indication for hysterectomy worldwide and they pose a significant socio-economic burden [2]. Approximately 10% of leiomyomas can be distinguished from “conventional” leiomyomas, as they display variant histopathology or distinct growth patterns [3]. Common histopathological variants include cellular leiomyomas, leiomyomas with bizarre nuclei, and mitotically active leiomyomas. Although these display some histopathological features associated with malignancy, they are considered clinically benign [3].

Recent studies have revealed the existence of various molecular leiomyoma subtypes. Indeed, 80–90% of leiomyomas harbor one of three genetic changes: a hotspot mutation in mediator complex subunit 12 (*MED12*), a chromosomal aberration resulting in upregulation of high mobility group AT-hook 2 (*HMGA2*), or biallelic loss of fumarate hydratase (*FH*) [4]. Pathogenic *FH* mutations may also be inherited, causing Hereditary Leiomyomatosis and Renal Cell Cancer (HLRCC) [5]. This syndrome is characterized by multiple uterine and cutaneous leiomyomas, as well as renal cell cancer of papillary type 2 histopathology. Mutations in genes encoding for members of the SRCAP histone-loading complex were recently discovered as a rare fourth molecular subtype [6]. While the known leiomyoma driver mutations have been discovered in most conventional tumors, a substantially larger proportion of leiomyoma variants lack them [7]. Cellular leiomyomas have been associated with 1p loss, but no well-established driver gene has been identified [8].

Uterine leiomyoma subtypes have been shown to display distinct global gene expression patterns [6, 9]. Leiomyomas of the *FH* subtype are characterized by activation of nuclear factor, erythroid 2 like 2 (NRF2) target genes, including upregulation of aldo-keto reductase family 1 member B10 (*AKR1B10*) and NAD(P)H quinone dehydrogenase 1 (*NQO1*) [9, 10]. NRF2 is an antioxidant defense transcription factor that is regulated by a Cullin-RING ligase (CRL) E3 ligase complex containing kelch like ECH associated protein 1 (KEAP1) as a substrate-recognition module and cullin 3 (CUL3) as a scaffold protein [11]. Excessive levels of fumarate result in protein succination, which is a chemical modification that occurs when fumarate reacts with cysteine residues to generate S-(2-succinyl)cysteine (2SC). Succination of critical cysteine residues within KEAP1 impairs its ability to direct NRF2 for ubiquitination and degradation by the proteasome, resulting in stabilization of *de novo* synthesized NRF2 [12, 13]. Oncogenic activation of the NRF2 pathway is a common feature in many cancer types [14]. While cancers frequently harbor loss-of-function mutations in *KEAP1* and *CUL3* or gain-of-function mutations in *NFE2L2* (encoding NRF2) itself [15], no such mutations have been detected in uterine leiomyomas. We and others have shown that overexpression of *AKR1B10* can function as a biomarker for FH-deficiency and NRF2 activation in uterine leiomyomas and renal cell cancer [9]. Indeed, we recently analyzed publicly available leiomyoma data and reclassified two leiomyomas as FH-deficient partly based on the expression of *AKR1B10* [16]. Using FH-deficient tumors derived from HLRCC patients and sporadic leiomyomas with biallelic loss of *FH*, we recently showed that AKR1B10 immunohistochemistry can act as a biomarker on protein-level as well [17]. In this study, we present a novel uterine leiomyoma subtype that is FH-proficient but displays AKR1B10 protein expression. We have integrated next-generation sequencing with gene expression profiling and discovered candidate driver mutations for these tumors. We have also evaluated the feasibility of AKR1B10 expression in detecting NRF2 activated tumors.

## Results

### Immunohistochemistry reveals AKR1B10 expression in FH-proficient leiomyomas

**Fig. 1:**
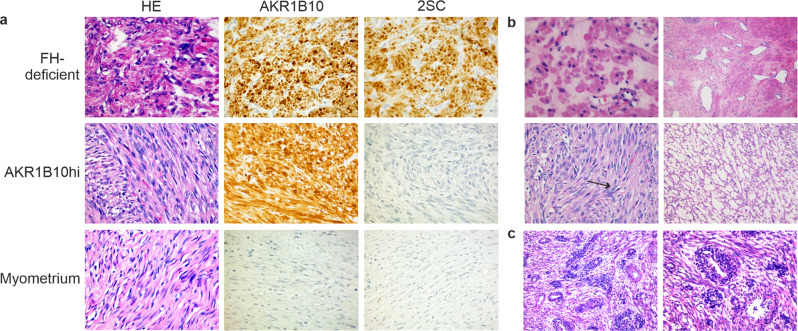
Immunohistochemistry and histopathological evaluation reveal AKR1B10 expression and specific morphological features in FH-proficient leiomyomas. **a** 2SC and AKR1B10 antibodies can both act as biomarkers for FH-deficiency. Immunohistochemistry showed strong staining with both antibodies in FH-deficient tumors. It also revealed a subgroup of tumors positive only for AKR1B10 (AKR1B10hi). Histopathological evaluation of the AKR1B10hi tumors showed increased cellularity in most of them. Myometrium was negative for both AKR1B10 and 2SC. **b** Half of the AKR1B10hi tumors showed two or more features associated with FH-deficiency [37], including eosinophilic inclusions (top left), staghorn vasculature (top right), eosinophilic nucleoli and nuclear atypia (bottom left), and alveolar edema (bottom right). Example of eosinophilic nucleoli is marked with an arrow. **c** Half of the AKR1B10hi samples displayed perivascular hypercellularity, a feature not associated with leiomyomas previously. Magnification 100× (left) and 200× (right).

To evaluate the expression of AKR1B10 in histopathological leiomyoma variants, we screened 141 FFPE variant tumors by immunohistochemistry. This sample set has been previously examined for defects in *MED12* by direct sequencing and alterations affecting HMGA2 or FH by immunohistochemistry [7, 18, 19]. As expected, all twelve FH-deficient leiomyomas displayed AKR1B10 expression (Supplementary Table 1 and Fig. 1a). In addition, we identified AKR1B10 expression in nine leiomyomas with no indication of FH-deficiency or alterations affecting *MED12* or HMGA2 (Table 1 and Fig. 1a). To study this further, we analyzed another previously examined dataset of 360 unselected leiomyomas [20] and discovered three additional tumors with AKR1B10 expression and no known driver defects. These twelve tumors are hereafter referred to as AKR1B10hi leiomyomas.

**Table 1:** Immunohistochemistry of 498 uterine leiomyomas reveal AKR1B10 expression in 12 FH-proficient tumors (AKR1B10hi).

Histopathology	*N*	AKR1B10hi	%	*p*-value**
Conventional	348	2	0.6	
Variant	150	10	6.7	0.0002
Cellular^a^	97	10	10.3	0.00001
Bizarre nuclei	32	0	0	1
Mitotically active	19	0	0	1
Epitheloid	1	0	0	1
Lipoleiomyoma	1	0	0	1

^a^Cellular leiomyomas included 12 tumors with also mitotic activity and two tumors with also bizarre nuclei. Two tumors with mitotic activity and two tumors with bizarre nuclei were AKR1B10hi tumors.

**p-values have been calculated with Fisher’s exact test. Frequency of AKR1B10hi in variant tumors has been compared to tumors with conventional histopathology.

### AKR1B10hi leiomyomas display distinct morphological features

Three uterine leiomyomas were included in both previously mentioned datasets and the total sample series hence consisted of 498 tumors, 348 with conventional and 150 with variant histopathology (Supplementary Table 1). Of the twelve AKR1B10hi samples, two had been diagnosed as conventional leiomyomas and ten displayed variant histopathology as defined by WHO classification [3] (Table 1). Interestingly, all ten variant tumors displayed increased cellularity. Six of the tumors showed cellularity as the only main histopathological feature, while two tumors displayed mitotic activity and two other tumors displayed bizarre nuclei as well. Overall, AKR1B10hi tumors were thus significantly over-represented in leiomyoma variants (*p*-value = 0.0002, two-sided Fisher’s exact test) and even more significantly over-represented in cellular leiomyomas (*p*-value = 0.00001, two-sided Fisher’s exact test) compared to conventional tumors. Thorough re-evaluation of the twelve AKR1B10hi tumors revealed that six of the tumors displayed at least two histopathological characteristics typically associated with FH-deficiency (Supplementary Table 2 and Fig. 1b). In addition, six tumors showed perivascular hypercellularity, a feature not usually found in uterine leiomyomas (Fig. 1c).

### Genes involved in neddylation of the Cullin 3-RING E3 ligase are recurrently mutated in AKR1B10hi leiomyomas

To identify candidate driver mutations and somatic copy number alterations (SCNA) in AKR1B10hi samples, we performed whole-exome and/or whole-genome sequencing with the twelve AKR1B10hi tumors and eight corresponding normal tissue samples. One exome tumor-normal pair was only analyzed for SCNAs as it displayed low coverage and poor sequencing quality. As expected, we detected no driver changes in *FH*, *MED12*, or *HMGA2*. We identified five genes that were mutated in more than one sample (Supplementary Table 3). Interestingly, two of the recurrently mutated genes encode for proteins involved in neddylation of the Cullin 3-RING E3 ligase; we identified three samples with a mutation in ubiquitin conjugating enzyme E2 M (*UBE2M*) and two samples with a mutation in NEDD8 ubiquitin like modifier (*NEDD8*) (Table 2 and Supplementary Fig. 1). Sanger sequencing revealed one additional *UBE2M* mutation in the tumor with poor exome-sequencing quality. These findings prompted us to search for other mutations related to neddylation among the non-recurrently mutated genes. We identified one sample with a mutation in the NEDD8-binding domain of Cullin 3 (*CUL3*). We then searched for additional mutations in an in-house exome sequencing dataset of seven fresh frozen leiomyomas. This revealed two mutations in NEDD8 activating enzyme E1 subunit 1 (*NAE1*) in one leiomyoma, which was negative for the known leiomyoma driver alterations. Histopathological evaluation of the corresponding FFPE sample revealed cellular histopathology and perivascular hypercellularity. In addition, immunohistochemistry showed strong staining for AKR1B10 adding this sample to the AKR1B10hi group. None of the identified neddylation-associated mutations were present in The Genome Aggregation Database (gnomAD). All variants were either truncating or predicted deleterious with a Combined Annotation-Dependent Depletion (CADD) value of over 20 or a Genomic Evolutionary Rate Profiling (GERP) value of over 2 [21, 22]. All mutations were validated by Sanger sequencing and confirmed as somatic where normal tissue was available (Supplementary Fig. 2).

**Table 2:** Candidate driver mutations in AKR1B10hi tumors.

Sample	Gene	Mutation	SCNA	Confirmed somatic	Prediction score
1301_1_S1	*UBE2M*	c.97C > T, p.(Arg33Trp)	Deletion	Yes	CADD: 28
1370_1_S1	*UBE2M*	c.281A > T, p.(Lys94Met)	Deletion	Yes	CADD: 28.7
1364_1_S1	*UBE2M*	c.298dupT, p.(Tyr100Leufs*6)	Deletion	Yes	NA
1356_1_S1	*UBE2M*	c.348_368delinsTCCG, p.(Arg116Serfs*40)	CN-LOH	Yes	NA
1367_1_S1	*UBE2M*	—	Deletion	—	—
1285_1_S1	*NEDD8*	c.8T > C, p.(Ile3Thr)	Deletion	NA	CADD: 31
1354_1_S1	*NEDD8*	c.44T > C, p.(Ile15Thr)	Deletion	Yes	CADD: 26.8
1593_1_S1	*NEDD8*	—	Deletion	—	—
1298_1_S2	*CUL3*	c.2222_2224del, p.(Ile741del)	Deletion	Yes	GERP: 5.4
1055_1_S1	*NAE1*	c.1422del, p.(Asp476Metfs*33)	—	NA	NA
		c.740T > A, p.(Ile247Asn)			CADD: 29.4

*SCNA* somatic copy number alteration; *CN-LOH* copy-neutral loss of heterozygosity, *NA* not analyzed (germline sample not available or in silico analysis not applicable).

Three AKR1B10hi samples (1308_1_S1, 1608_1_S4, and 1626_1_S2) did not harbor any candidate driver changes related to neddylation.

A combined annotation-dependent depletion (CADD) value of over 20 indicates a deleterious change.

A genomic evolutionary rate profiling (GERP) value of over 2 indicates high evolutionary conservation.

**Fig. 2:**
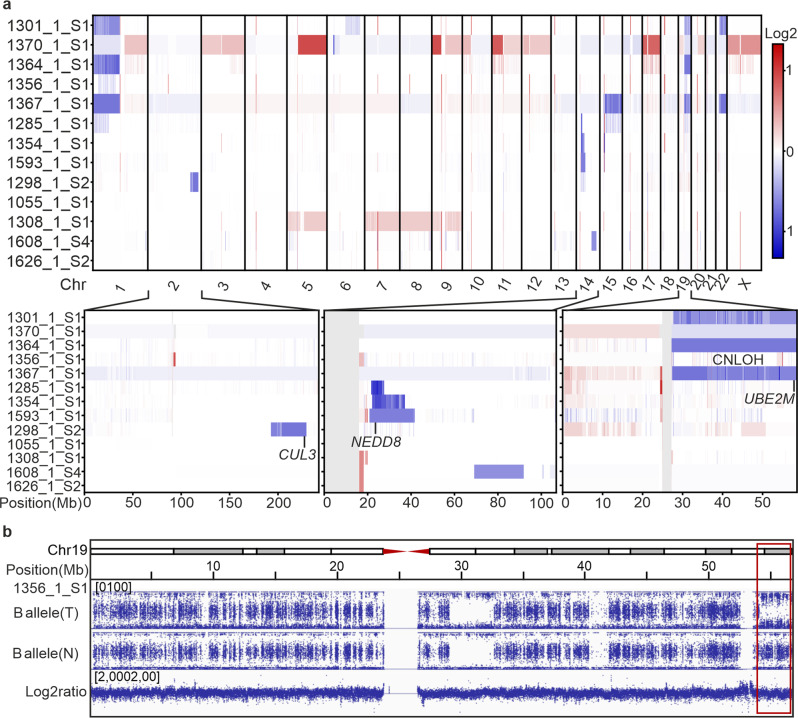
Somatic copy number alterations in AKR1B10hi leiomyomas. **a** AKR1B10hi leiomyomas displayed relatively stable chromosomal profiles. Recurrent loss of the 19q-arm was seen in four AKR1B10hi leiomyomas and three of these deletions overlapped with a gene-level mutation in *UBE2M*. An interstitial 14q deletion was seen in three leiomyomas and two of these deletions overlapped with a gene-level mutation in *NEDD8*. Only one sample harbored a 2q deletion, and this deletion overlapped with a gene-level mutation in *CUL3*. **b** One sample with a gene-level mutation in *UBE2M* displayed copy-neutral loss of heterozygosity (CN-LOH) at the terminal q-arm of chromosome 19.

Somatic copy number alteration analysis of all thirteen AKR1B10hi tumors revealed that most chromosomes were relatively stable (Fig. 2a). However, we did identify recurrent whole-arm and terminal losses on chromosomes 1p, 19q, and 22q, as well as three interstitial deletions affecting 14q. In addition, we identified one sample with a terminal copy-neutral loss of heterozygosity (CN-LOH) event at 19q (Fig. 2b). The 19q and 14q alterations frequently overlapped with the gene-level mutations, resulting in biallelic loss of *UBE2M* on 19q13.43 and biallelic loss of *NEDD8* on 14q12. Alterations affecting 1p, 19q, and 22q co-occurred with each other, whereas the interstitial deletions in 14q were mutually exclusive with the former changes. Only one sample displayed an interstitial deletion in 2q, resulting in biallelic loss of *CUL3* (Fig. 2a). We identified no large-scale chromosomal alterations in the sample that harbored two *NAE1* mutations. Large-scale amplified regions were seen in only two samples, including a shared amplification of the 5q-arm and whole-chromosome amplification of chromosome 9 (Fig. 2a).

### 3′RNA sequencing confirms activation of the NRF2 pathway in AKR1B10hi leiomyomas

**Fig. 3:**
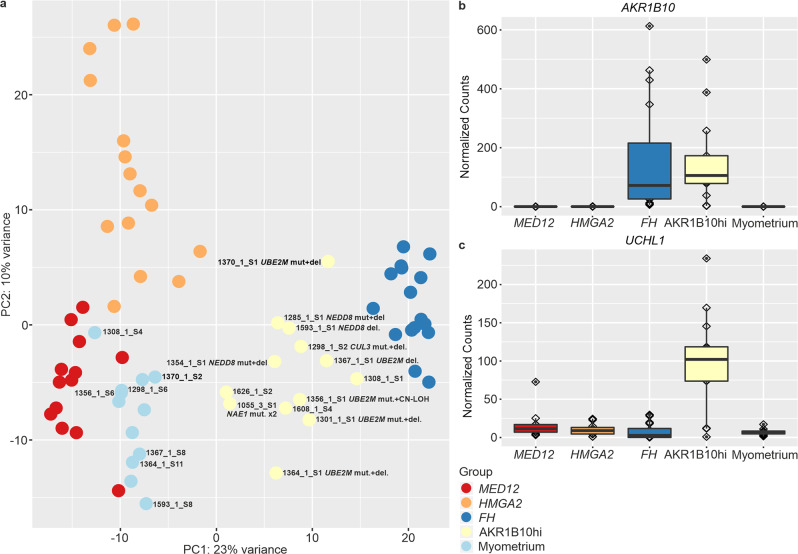
Principal component analysis and differentially expressed genes in AKR1B10hi leiomyomas. **a** Thirteen AKR1B10hi leiomyomas and seven corresponding myometrium samples were analyzed together with a previously published dataset of 44 leiomyoma and 5 myometrium samples. Principal component analysis revealed that the AKR1B10hi samples cluster separately from leiomyomas of the *MED12*, *HMGA2*, and *FH* subtypes. Samples analyzed in this study are marked with sample identifiers. **b** *AKR1B10* was significantly upregulated in AKR1B10hi and FH-deficient leiomyomas, but not in the other leiomyoma subtypes. **c** Deubiquitination gene *UCHL1* was among the most uniquely upregulated genes in AKR1B10hi leiomyomas.

To explore the gene expression pattern of AKR1B10hi leiomyomas, we performed 3′RNA sequencing with thirteen AKR1B10hi leiomyomas and seven corresponding myometrium samples. We analyzed these samples together with a previously published dataset of 44 leiomyomas [16]. Principal component analysis revealed that the AKR1B10hi samples clustered separately from the other three established leiomyoma subtypes, but close to leiomyomas of the *FH* subtype (Fig. 3a).

Differential expression analysis comparing AKR1B10hi samples against the myometrium controls revealed 1014 differentially expressed genes (*q*-value < 0.05; |FC | > 2, Supplementary Table 4), with *AKR1B10* being the second most significant one (FC = 34.54, *q*-value = 4.68 × 10^−25^, Fig. 3b). Pathway enrichment analysis confirmed significant dysregulation of the NRF2 pathway in this novel leiomyoma subtype (*q*-value = 3.29 × 10^−03^; Supplementary Table 5), including upregulation of the well-established NRF2 target gene *NQO1* (FC = 5.58, *q*-value = 3.07 × 10^−12^). Immunohistochemistry confirmed overexpression of NQO1 in 11 AKR1B10hi samples (Supplementary Fig. 3). Two AKR1B10hi samples failed in the analysis as no NQO1 expression was detected in either the internal control (endothelial) cells nor in the smooth muscle cells. To find uniquely expressed genes, we compared AKR1B10hi leiomyomas against the other leiomyoma subtypes and myometrium samples and identified 585 significant genes (Supplementary Table 6). Ubiquitin C-terminal hydrolase L1 (*UCHL1*) was the third most significant gene (FC = 6.11, *q*-value = 1.09 × 10^−11^, Supplementary Table 6 and Fig. 3c).

### AKR1B10 is a robust biomarker for NRF2 activation across different malignancies

To determine the feasibility of AKR1B10 as a biomarker for NRF2 activation across different malignancies, we exploited The Cancer Genome Atlas (TCGA) and Cancer Cell Line Encyclopedia (CCLE) multilevel data. First, we utilized receiver-operating characteristic (ROC) analysis to investigate how *AKR1B10* mRNA expression is associated with hotspot and truncating mutations in *KEAP1* and hotspot mutations in *NFE2L2* in all TCGA samples. *AKR1B10* mRNA exhibited robust discrimination of NRF2 activation with an area under the ROC curve of 0.91 (Supplementary Fig. 4a). This was further validated at the protein level, as AKR1B10 protein expression correlated with the mRNA in CCLE data (*r* = 0.82, *p* < 0.001, Supplementary Fig. 4b).

### NRF2 activation through mutations in neddylation-associated genes is rare in cancers

We utilized multilevel TCGA data to investigate whether somatic mutations in *UBE2M*, *NAE1*, *NEDD8*, or the NEDD8-binding domain of *CUL3* can be found in cancers with NRF2 activation. Based on the ROC analysis, we defined tumors with an activated NRF2 by utilizing the true positive rate of over 0.8 for AKR1B10 expression. We then removed samples with a somatic mutation in *NFE2L2*, *KEAP1*, *FH*, or *CUL3* (excluding the NEDD8-binding domain). This revealed 11 samples across six different cancer types with a nonsynonymous mutation in a neddylation-associated gene: four with a *UBE2M* mutation, five with a *NAE1* mutation, one with a *NEDD8* mutation, and one with a mutation in the NEDD8-binding domain of *CUL3* (Supplementary Figs. 1 and 4c).

### Inhibition of neddylation in melanoma cells results in activation of the NRF2 pathway

Using publicly available microarray data, we explored the global gene expression pattern of melanoma cells that had been treated with the NEDD8-activating enzyme inhibitor MLN4924 for up to 24 h. Gene set enrichment analysis (GSEA) revealed a significant enrichment of gene sets related to the NRF2 pathway (Supplementary Fig. 5a). Furthermore, we observed a gradual increase in *AKR1B10* expression with a peak fold-change of 4.45 in cells that had been treated with MLN4294 compared to controls (Supplementary Fig. 5b). According to fold-change ranking at the 24 h time point, *AKR1B10* was the 27th most upregulated gene among 19421 genes examined on the array.

## Discussion

Uterine leiomyomas are benign smooth muscle tumors that can be classified into at least three molecular subtypes, reflecting mutations in either *MED12*, *HMGA2*, or *FH* [4]. We have previously shown that each defect results in a distinct global gene expression pattern [9]. Leiomyomas of the *FH* subtype are characterized by activation of the NRF2 pathway, including upregulation of the NRF2 target gene *AKR1B10* [9]. In this study, we found AKR1B10 expression in a subset of leiomyomas with no *FH* mutations. These tumors were also negative for *MED12* and *HMGA2* defects, as well as for mutations in genes encoding for proteins of the SRCAP complex, another recently discovered leiomyoma subtype [6]. This suggests that these AKR1B10 overexpressing tumors represent a novel leiomyoma subtype.

Using next-generation sequencing, we identified biallelic mutations in key genes involved in neddylation of the Cullin 3-RING E3 ligase [23, 24]. Neddylation is analogous to ubiquitination and the process by which NEDD8 is conjugated to target proteins for degradation [25]. NEDD8 is a ubiquitin-like protein that activates the largest family of ubiquitin E3 ligases, the Cullin–RING ligases [26]. Neddylation is mediated by a NEDD8-activating enzyme E1 (NAE, a heterodimer consisting of NAE1 or UBA3), a NEDD8-conjugating enzyme E2 (UBE2M or UBE2F), and a NEDD8 ligase E3 (including the KEAP1/CUL3/RBX1 E3-ubiquitin ligase complex) [26]. Here, we identified mutually exclusive mutations in *NEDD8*, *NAE1*, and *UBE2M*. All mutations were either truncating or predicted deleterious and frequently accompanied by loss of heterozygosity, suggesting that they result in biallelic inactivation and defective neddylation. We were unable to find a second mutation in *UBE2M* in one tumor with a 19q loss and in *NEDD8* in one tumor with an interstitial 14q deletion. However, deletions in 19q and 14q were mutually exclusive in all tumors studied, suggesting that they indeed target *UBE2M* and *NEDD8*, respectively. UBE2M is specifically involved in neddylation of Cullins 1–4 and interestingly, we also identified a 3 bp deletion in the NEDD8-binding domain of *CUL3*, suggesting that the identified mutations specifically disrupt neddylation of the Cullin 3-RING E3 ligase [27]. Indeed, a Cul3K712R mutant in the same domain has been shown to result in reduced binding of NEDD8 to CUL3 [28].

**Fig. 4:**
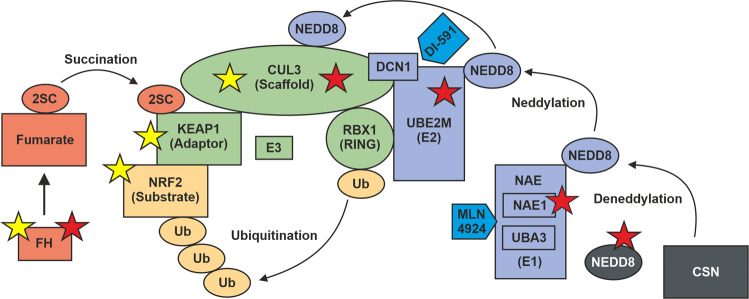
Schematic illustration of mechanisms leading to oncogenic NRF2 activation. NRF2 is, under basal conditions, ubiquitinated by the KEAP1/CUL3/RBX1 E3-ubiquitin ligase complex. Neddylation is required for the activation of cullin-RING ubiquitin ligases (CRL). The COP9 signalosome (CSN) is responsible for deneddylation of CRL. KEAP1 plays a central role in regulating the activity of NRF2. In cancers, the interaction between the E3 subunits can be disrupted by specific mutations in *NFE2L2* (encoding NRF2) or by biallelic loss of *KEAP1* or *CUL3* (yellow stars). The interaction between NRF2 and KEAP1 can also be disrupted by protein succination of critical cysteine residues within KEAP1 as a result of fumarate accumulation. Oncogenic activation of the NRF2 pathway can be seen in uterine leiomyomas that harbor biallelic loss of *FH* or biallelic loss of key members involved in the neddylation pathway (red stars). Finally, inhibition of neddylation by MLN4924 or DI-591 results in activation of NRF2.

Gain-of-function mutations in *NFE2L2*, loss-of-function mutations in key members of the KEAP1/CUL3/RBX1 E3-ubiquitin ligase complex, and succination of KEAP1 have proven to activate the NRF2 pathway in many cancer types [14]. Our results suggest that defective neddylation represents a novel mechanism for oncogenic NRF2 activation (Fig. 4). Using 3′RNA sequencing and immunohistochemistry of NQO1, we confirmed a significant dysregulation of the NRF2 pathway in AKR1B10hi tumors. To our knowledge, mutations in neddylation-associated genes have not been previously identified as potential drivers of tumorigenesis. Using multilevel TCGA and CCLE data, we showed that *AKR1B10* expression is a robust biomarker for NRF2 activation across cancers. By utilizing *AKR1B10* as a biomarker for NRF2 activation, we found only 11 samples with NRF2 activation and a candidate mutation in *UBE2M*, *NAE1*, *NEDD8*, or the NEDD8-binding domain of *CUL3*. This suggests that defective neddylation is not a common mechanism driving NRF2 activation in cancers.

MLN4924 is a potent and selective small-molecule inhibitor of NAE1 [29]. Functional studies have shown that inhibiting neddylation by the MLN4924 inhibitor results in stabilization of the NRF2 protein [29, 30]. Using publicly available expression data of melanoma cells treated with the MLN4924 inhibitor, we confirmed significant upregulation of NRF2 target genes, including *AKR1B10* as one of the most significantly upregulated genes. In addition, defective in cullin neddylation 1 (DCN1) promotes neddylation of Cullin-RING ubiquitin ligases by interacting with UBE2M [31, 32]. DCN1 is more critical in neddylation of cullin 3 than other cullin family members. DI-591 is another small-molecule inhibitor that binds to DCN1 and selectively converts cellular cullin 3 into an un-neddylated inactive form [31]. Furthermore, knockdown of DCN1 has been shown to result in accumulation of NRF2. These data support that defective neddylation leads to oncogenic NRF2 activation and strengthen the notion of neddylation-associated mutations as potential drivers in tumorigenesis.

The AKR1B10hi tumors displayed a global expression pattern distinct from the other established leiomyoma subtypes. In contrast to FH-deficient leiomyomas that also display NRF2 activation, but presumably functional neddylation, we identified the deubiquitin gene *UCHL1* as one of the most uniquely upregulated genes in AKR1B10hi tumors. *UCHL1* encodes for an enzyme called ubiquitin carboxyl-terminal esterase L1 (UCH-L1) that interacts with the COPS5 subunit of the COP9 signalosome (CSN) [33]. CSN is a protein complex that deactivates Cullin-RING ubiquitin ligases by catalyzing the hydrolysis of NEDD8 from the cullin subunit [34]. UCH-L1 has a high affinity for both ubiquitin and NEDD8, but it can only hydrolyze ubiquitin, unlike its more widely distributed homolog UCH-L3 [35]. The possibility of UCH-L1 exerting an effect through binding to and/or regulating NEDD8 has been proposed, but remains to be fully explored [36]. It is tempting to speculate that upregulation of *UCHL1* may be a consequence of defective neddylation in these tumors.

Approximately 10% of leiomyomas display variant histopathology with increased cellularity being the most common feature. Histopathological features associated with FH-deficiency are typically seen in leiomyomas with bizarre nuclei, but rarely in leiomyomas with cellular histopathology [37, 38]. Here, we discovered more than one FH-associated feature in six AKR1B10hi tumors: in five cellular leiomyomas and in one leiomyoma with conventional histopathology. This suggests that morphological features typically seen in FH-deficient tumors may be related to activation of the NRF2 pathway itself, and not with loss of *FH*. The majority of AKR1B10hi leiomyomas also showed perivascular hypercellularity, a feature not typically seen in uterine leiomyomas.

Cellular leiomyomas have previously been associated with the loss of 1p, and these tumors display some gene expression patterns seen in leiomyosarcomas [39]. Multiple studies have reported 1p loss to be frequently accompanied by 19q loss in leiomyomas, leiomyosarcomas, and benign metastasizing leiomyoma [8, 40–43]. Indeed, all the samples with 1p loss also displayed 19q loss. Interestingly, a recent study reported 1p/19q co-deletions in a subset of leiomyosarcomas lacking mutations in *TP53* and *RB1* [44]. In addition, 1p/19q co-deletions are common in oligodendrogliomas that harbor a mutation in isocitrate dehydrogenase 1 (*IDH1*) [45]. *IDH1* and *FH* both encode for key enzymes of the citric acid cycle [46, 47]. We have previously shown *UBE4B* to be the most significantly downregulated gene on the 1p arm in leiomyomas harboring a 1p deletion [7]. *UBE4B* encodes for an additional conjugation factor, E4, which is involved in multiubiquitin chain assembly [48, 49]. It is tempting to speculate that the 1p/19q co-deletion may simultaneously target *UBE2M* on 19q13.43 and *UBE4B* on 1p36.22.

In this study, we have identified a novel uterine leiomyoma subtype with biallelic mutations in genes involved in neddylation of the Cullin 3-RING E3 ligase. Most mutated tumors displayed specific morphological features and a distinct gene expression profile characterized by activation of the NRF2 pathway. Compatible with functional studies on neddylation, our observations indicate disrupted neddylation as a novel mechanism leading to oncogenic NRF2 activation in human tumors. Development of NRF2 inhibitors has recently emerged as a promising anticancer strategy [50]. Overall, molecular stratification of uterine leiomyomas paves the way for more personalized treatment, as these very common tumors are still often considered as a single disease entity.

## Materials and methods

### Study material

The study has been approved by The Ethics Review Board of Hospital District of Helsinki and Uusimaa, Helsinki, Finland (HUS; 88/13/03/03/2015). Samples were collected with written informed consent from the patients or with authorization from the National Supervisory Authority of Welfare and Health (Valvira). The study material consisted of archival formalin-fixed paraffin-embedded (FFPE) tissue samples and corresponding hematoxylin-eosin (HE) stained slides that were obtained from the Department of Pathology, Helsinki University Hospital, Helsinki, Finland. Histopathological examination was performed by a pathologist specialized in gynecological pathology (RB or AP). The initial discovery set consisted of 141 leiomyomas with variant histopathology [18]. The validation set included 360 unselected leiomyomas, of which 12 displayed variant histopathology [20]. Three samples in the validation set were also included in the discovery set and therefore omitted from the latter cohort. The total sample series thus included 498 tumor samples, of which 348 displayed conventional and 150 variant histopathology. The status of *MED12*, HMGA2, and FH has been determined in previous studies [7, 18–20]. See Supplementary Table 1 for an overview of the samples. Normal tissue samples (myometrium or fallopian tube) were available for eight of the twelve patients with an AKR1B10hi tumor. In addition, an in-house exome-sequencing dataset of seven fresh frozen leiomyomas and five corresponding normal tissue samples was examined. FFPE tissue and corresponding HE-stained slides were obtained from one patient (1055) whose tumor showed two *NAE1* mutations.

### Immunohistochemistry

AKR1B10 immunohistochemistry was performed using tissue microarrays (TMA). When the sample showed positive staining in the TMA analysis or if the result was unclear, the corresponding whole tissue section was analyzed. The FH status for the variant samples has been previously determined with a 2SC antibody [7] or with an FH antibody [19]. In this study, samples previously stained only with the FH antibody were stained with the 2SC antibody for consistency. In brief, after deparaffination, heat-introduced antigen retrieval, and peroxidase blocking, incubation with the primary antibody AKR1B10 (1:300, H00057016-M01, Abnova, Taipei, Taiwan), 2SC (1:1000, crb2005017d, Discovery Antibodies, Billingham, UK), or NQO1 (1:500, sc-32793, Santa Cruz Biotechnology, Inc., Dallas, TX, USA) was performed overnight. Post antibody blocking (Immunologic BV, Duiven, Netherlands: post antibody blocking for bright vision plus) was followed by incubation with a secondary poly-HRP antibody (ImmunoLogic: Poly-HRP-GAM/R/R IgG). DAB Quanto (Thermo Fisher Scientific, Waltham, MA, USA) system was used to detect the expression levels. A pathologist (RB) evaluated the expression levels using a three-grade scoring system: ++ = strong staining, + = weak staining, and – = no staining. Both strong and weak staining were considered positive.

### DNA and RNA extraction

Representative areas of tumor or normal tissue were marked on HE slides by a pathologist. Depending on the marking, DNA was extracted from the whole FFPE tissue sections or from six representative 0.8 mm cores with the ReliaPrep FFPE gDNA Miniprep System (Promega, Madison, WI, USA). DNA from FFPE samples that were selected for whole-exome (WES) and whole-genome sequencing (WGS) were extracted using the standard phenol-chloroform method. Fresh frozen tissue samples had been extracted with the FastDNA Spin Kit (MP Biomedicals, Santa Ana, CA, USA).

Total RNA was extracted and purified from macrodissected sections using the RNeasy® FFPE Kit (QIAGEN, Hilden, Germany) and the deparaffinization solution (QIAGEN) according to the manufacturer’s instructions. The concentration and purity of the extracted RNA were examined using the LabChip GX Touch HT RNA Assay Reagent Kit (PerkinElmer, Waltham, MA, USA) and the Qubit RNA BR kit (Thermo Fisher Scientific, Waltham, MA, USA). Genomic DNA contamination was measured with the Qubit DNA BR kit (Thermo Fisher Scientific).

### Whole-exome and whole-genome sequencing

Tissue samples from 13 patients were subjected to WES and/or WGS. WES was performed with twelve tumors and two corresponding normal tissue samples at Biomedicum Functional Genomics Unit (FuGU), Helsinki, Finland on the Illumina (Illumina, San Diego, CA, USA) NextSeq 500 platform using 2 × 75bp paired-end reads. These 14 samples were prepared using the KAPA Hyper Prep kit (Roche NimbleGen, Madison, WI, USA) and captured using the SeqCap EZ MedExome Kit (Roche). WGS was performed with nine tumors and six corresponding normal tissue samples at Beijing Genomics Institute (BGI) on the BGISEQ-500 platform using 2 × 150 bp or 2 × 100 paired-end reads. These samples were prepared using the KAPA Hyper Prep kit (Roche). See Supplementary Table 7 for an overview of the methods that were used for each sample. One WES tumor was not analyzed for point mutations and microindels as it displayed very low quality and coverage.

Adapter and read trimming were performed using Trimmomatic [51]. WGS samples that were sequenced using 150bp reads were trimmed to 100 bp in length. WES and WGS data were preprocessed according to Genome Analysis ToolKit 4 best practices [52]. In brief, the samples were aligned against the Genome Reference Consortium Human Build 38 genome using BWA-MEM [53], duplicate reads were removed using Mark Duplicates, and base quality score recalibration was performed using BaseRecalibrator.

Paired and non-paired somatic variant calling was performed using Mutect2 with default parameters [52]. FFPE artifacts were identified using LearnReadOrientationModel [52]. Identified variants were filtered against a panel of normals consisting of 48 exomes and 28 genomes, a panel of normals generated from the 1000 genomes project, and variants present in the Genome Aggregation Database (exomes and genomes v2.0.1 and v3) using Baseplayer [54]. Recurrent nonsynonymous variants and microindels with a sequencing depth of at least 16, an allelic count of at least 8, and an allelic fraction of 0.3 were evaluated further using BasePlayer and annotated using variant effect predictor [55].

Somatic copy number alterations (SCNA) were called using CNVkit with default parameters [56]. SCNA for WGS samples were called against a pooled normal generated using six corresponding normal tissue samples, and SCNA for WES samples were called against a pooled normal generated using five in-house FFPE normal tissue samples. SCNA for one fresh frozen sample were called against a pooled normal generated using eight in-house fresh frozen normal tissue samples. Heatmaps of SCNA were visualized with the -d option to de-emphasize low-amplitude segments. WGS calls were used for visualization, unless only WES data was available.

### Sanger sequencing

Mutations in *UBE2M*, *NEDD8*, *CUL3*, and *NAE1* were validated by direct Sanger sequencing. One tumor that failed in exome sequencing was screened for mutations in the coding regions of *UBE2M* and *NEDD8*. Primers and conditions are available upon request. Sequencing was performed using the Applied Biosystems (ABI) 3730 DNA Sequencer at the Institute for Molecular Medicine Finland (FIMM), Helsinki, Finland. Sequence graphs were analyzed and visually inspected using Mutation Surveyor (SoftGenetics, State College, PA, USA) and FinchTV (Geospiza, Inc, Seattle, WA, USA).

### 3′RNA sequencing

3′RNA sequencing was performed with 13 leiomyomas and 7 corresponding myometrium samples as previously described [16]. These 20 samples were analyzed together with a previously published dataset of 44 leiomyomas (13 leiomyomas with a *MED12* mutation, 15 with significant HMGA2 overexpression, and 16 with FH- deficiency) and 5 myometrium samples [16].

Dual-indexed mRNA libraries were prepared using QuantSeq 3′mRNA-Seq Library Prep Kit FWD (Lexogen Gmbh, Vienna, Austria) following the manufacturer’s protocol. Sequencing was performed at FIMM using the NovaSeq 6000 System (Illumina) with a read length of 2 × 101 base pairs and a minimum target coverage of 15 M reads for each library. FASTQ preprocessing was performed using the Integrated Data Analysis Pipeline version 2.3.1 FWD UMI (Lexogen Gmbh) implemented on the Bluebee® Genomics analysis platform. Briefly, the reads were trimmed with BBDuk, aligned to the Genome Reference Consortium human build 38 (GRCh38) reference genome using STAR [57], and counted using HTSeq [58]. Read counts of the technical replicates were merged. Raw read counts were normalized using DESeq2 [59]. Principal component analysis (PCA) and pairwise differential expression analysis were performed with DESeq2 on the Chipster platform [59, 60]. Pathway enrichment analysis of over-represented WikiPathways was performed using the WEB-based GEne SeT AnaLysis Toolkit [61].

### TCGA and CCLE data analysis

TCGA mRNA counts were downloaded from the Broad GDAC Firehose database (2016_01_28) [62]. TCGA mutational data was obtained from the NCI Genomic Data Commons (v0.2.8.) [63]. Read counts were TMM-normalized and voom-transformed using edgeR (v3.26.8). To evaluate the performance of *AKR1B10* expression as a classifier for NRF2 activation, we performed ROC analysis using ROCR (v1.0–11) [64]. Samples with truncating and well-established hotspot mutations in *NFE2L2* and *KEAP1* [65] served as positive controls for NRF2 activation. Identified mutations in *UBE2M*, *NAE1*, *NEDD8*, and *CUL3* were visualized using maftools (R-version 3.6.0) [66]. Pre-normalized CCLE mRNA and proteomic data was downloaded from DepMap [67, 68] and CCLE (20Q4) [69] databases.

### Gene expression microarray data analysis

Affymetrix GeneChip Human Genome U133 Plus 2.0 microarray data of melanoma cells that had been treated with MLN4924 or DMSO were obtained from the ArrayExpress database (accession: E-GEOD-30531). The data was normalized by the RMA method with affy (v1.62.0) R-package. Differential expression analysis was conducted using limma and EdgeR [70, 71]. Time course differential expression analysis was performed using limma. GSEA was performed with WikiPathways (Java GSEA v3.0) and the Singh NFE2L2 targets gene set, using MLN4924 treatment time as a continuous variable against DMSO treated samples [72, 73].

## Supplementary information

Supplementary Figures

Supplementary Tables

## Data Availability

All key findings are presented in the manuscript and supplementary files. The raw data are not publicly available due to compliance with the ethics approval and confidentiality agreements. Data may be obtained from the authors upon reasonable request when compatible with European General Data Protection Regulation (GDPR) and with the permission from the University of Helsinki.
