# Supplementary material for: A novel uterine leiomyoma subtype exhibits NRF2 activation and mutations in genes associated with neddylation of the Cullin 3-RING E3 ligase
Source: Oncogenesis. 2022 Sep 7;11(1):52. doi: 10.1038/s41389-022-00425-3 (PMC9448808; doi:10.1038/s41389-022-00425-3)
Supplement: Supplementary file 1 — Supplementary Figures [file 41389_2022_425_MOESM1_ESM.pdf]

Supplementary Figures

A novel uterine leiomyoma subtype exhibits NRF2 activation and mutations in genes associated with neddylation of the Cullin 3-RING E3 ligase

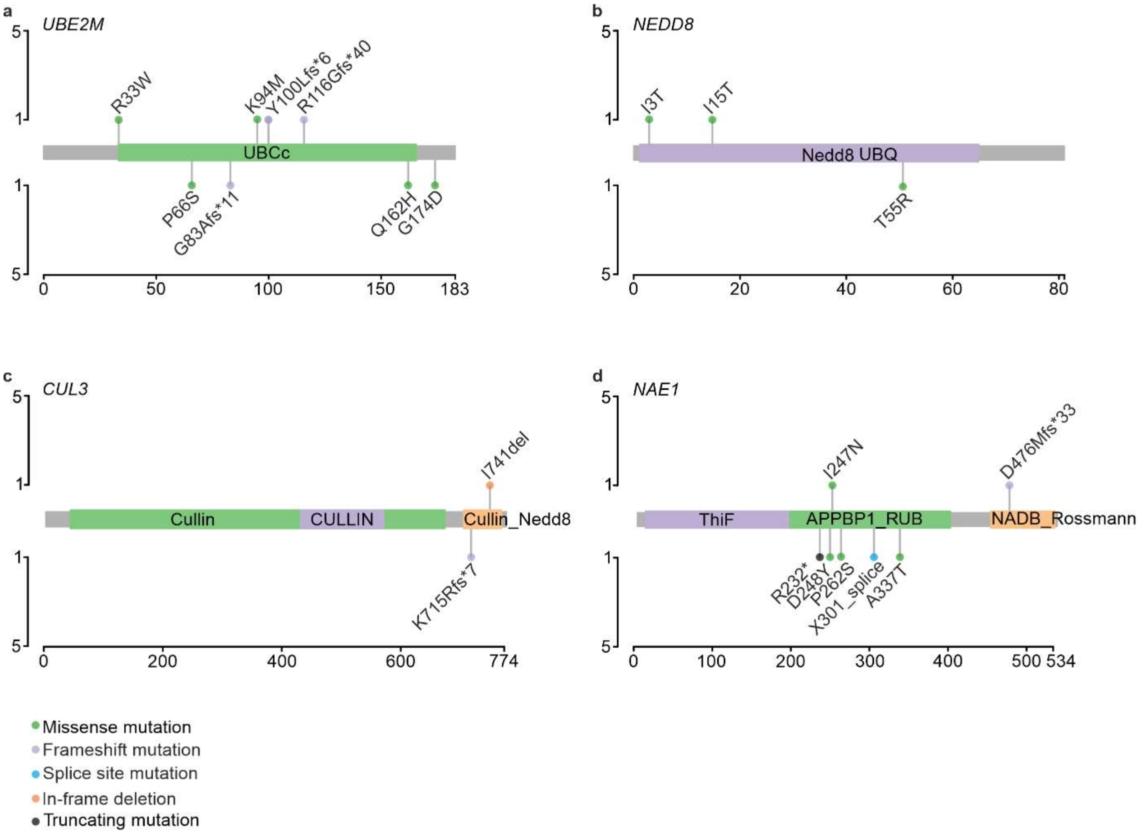

Supplementary Figure 1. Lollipop plots of mutations identified in *UBE2M*, *NEDD8*, *CUL3*, and *NAE1*. Mutations identified in **a)** *UBE2M*, **b)** *NEDD8*, **c)** NEDD8-binding domain of *CUL3*, and **d)** *NAE1* in uterine leiomyomas (facing up) and in TCGA data (facing down). From TCGA data, only tumors with NRF2 activation are included.

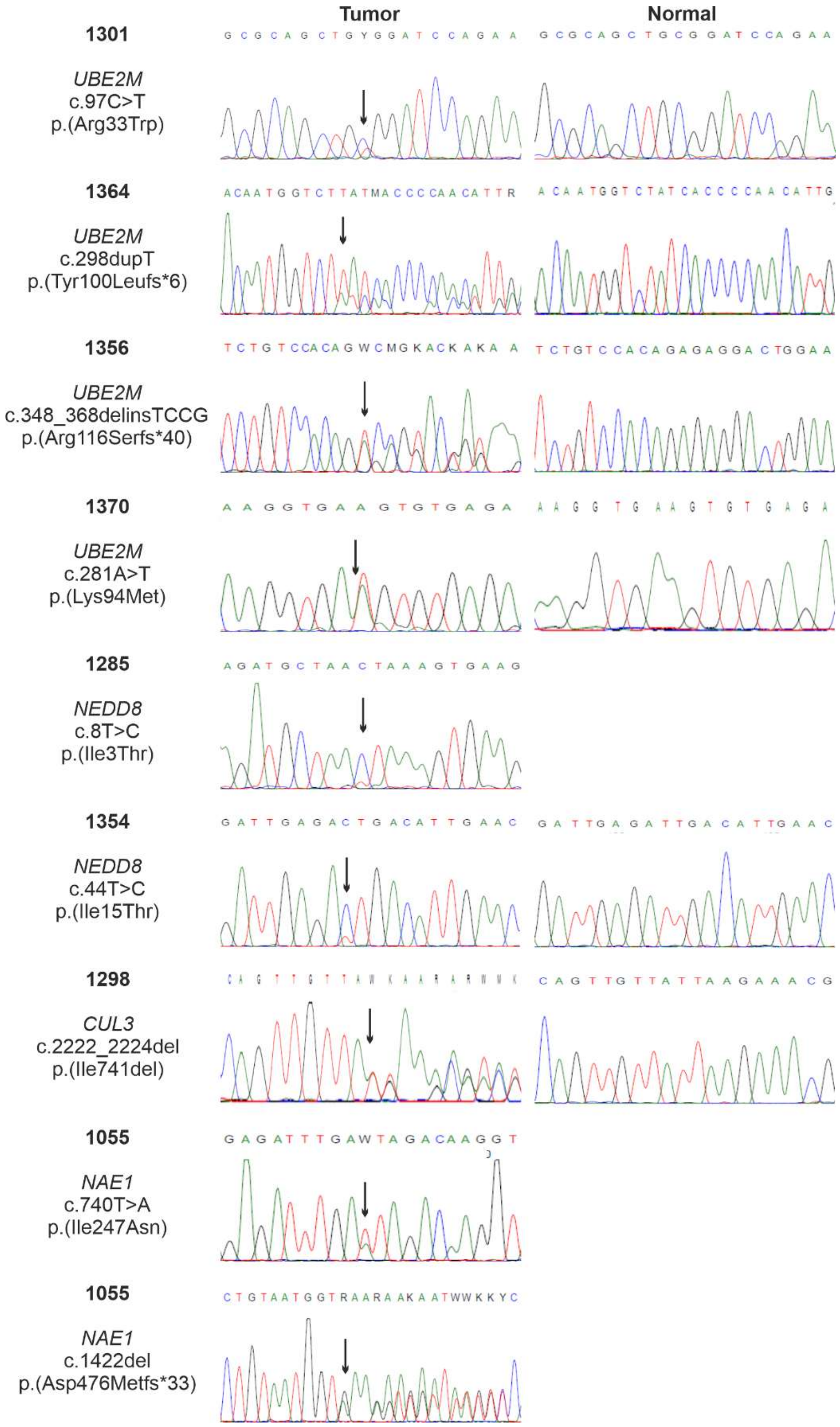

Supplementary Figure 2. Chromatograms of mutations identified in *UBE2M*, *NEDD8*, *CUL3*, and *NAE1*. Sanger sequencing confirmed mutations in *UBE2M*, *NEDD8*, *CUL3*, and *NAE1*. The mutations were confirmed somatic in all cases where normal tissue was available.

a

b

c

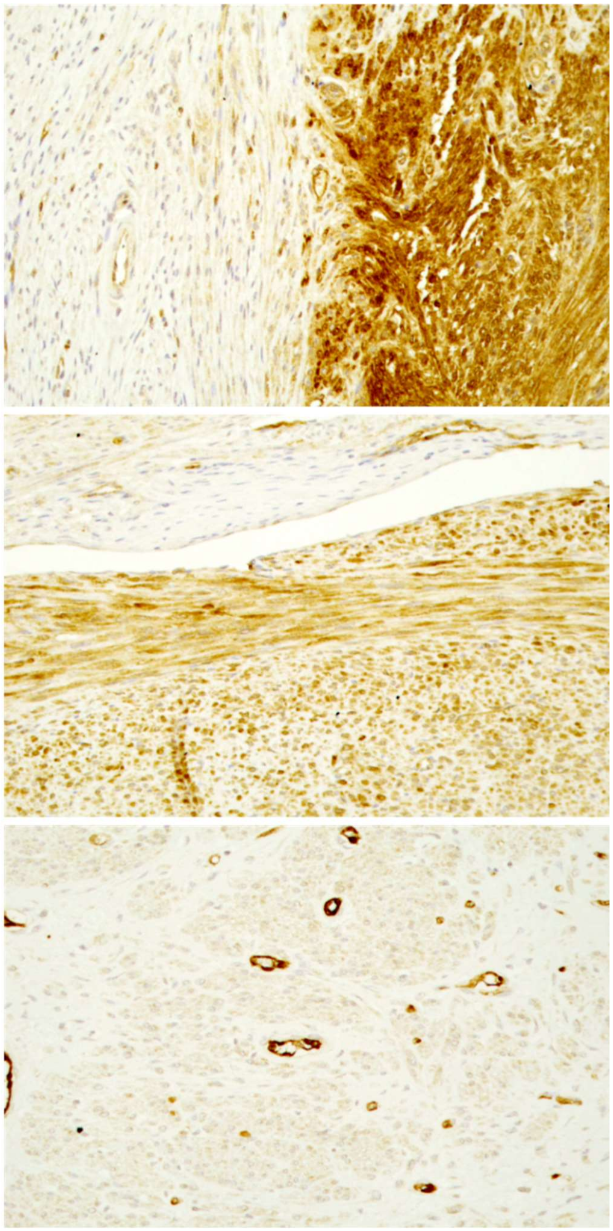

Supplementary Figure 3. Immunohistochemical analysis of the NRF2 target gene NQO1. Eleven out of thirteen AKR1B10hi samples were successfully stained and all eleven showed overexpression of NQO1. FH-deficient samples were used as positive controls and FH-proficient leiomyomas were used as negative controls. **a)** Representative AKR1B10hi sample showing NQO1 expression in smooth muscle tumor cells. **b)** Representative FH-deficient positive control sample showing NQO1 expression in smooth muscle tumor cells. **c)** Representative negative control sample showing NQO1 expression only in endothelial cells as an internal control (Magnification 200×).

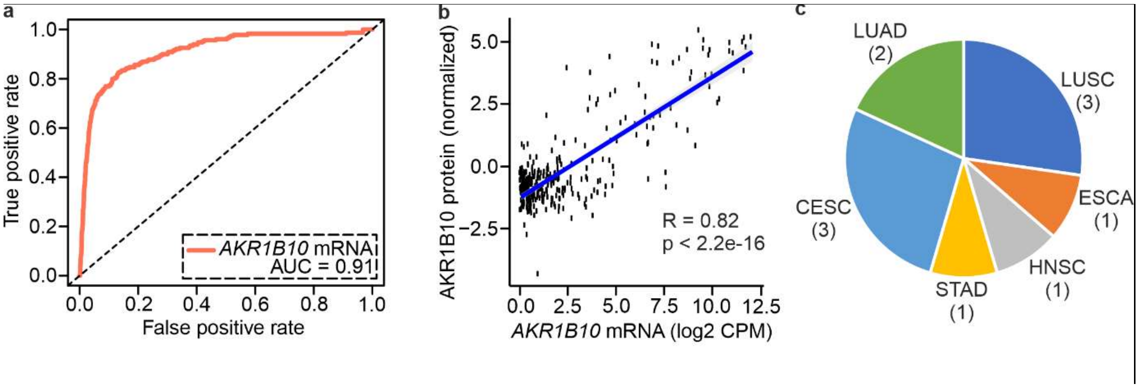

Supplementary Figure 4. Multilevel TCGA and CCLE data confirm that AKR1B10 is a robust biomarker for NRF2 activation and reveal that neddylation-associated mutations are rare in NRF2 activated cancers. a) ROC analysis showed that *AKR1B10* mRNA robustly discriminates NRF2 activation in TCGA data with an area under the curve (AUC) value of 0.91. **b)** In the CCLE data, *AKR1B10* mRNA correlated with AKR1B10 protein expression. **c)** Neddylation-associated mutations were discovered in 11 NRF2 activated cancers in TCGA data (ESCA: esophageal carcinoma; CESC: squamous cell carcinoma; STAD; stomach adenocarcinoma; LUSC: lung squamous cell carcinoma; LUAD: lung adenocarcinoma; HNSC head-neck squamous cell carcinoma). CPM: counts per million.

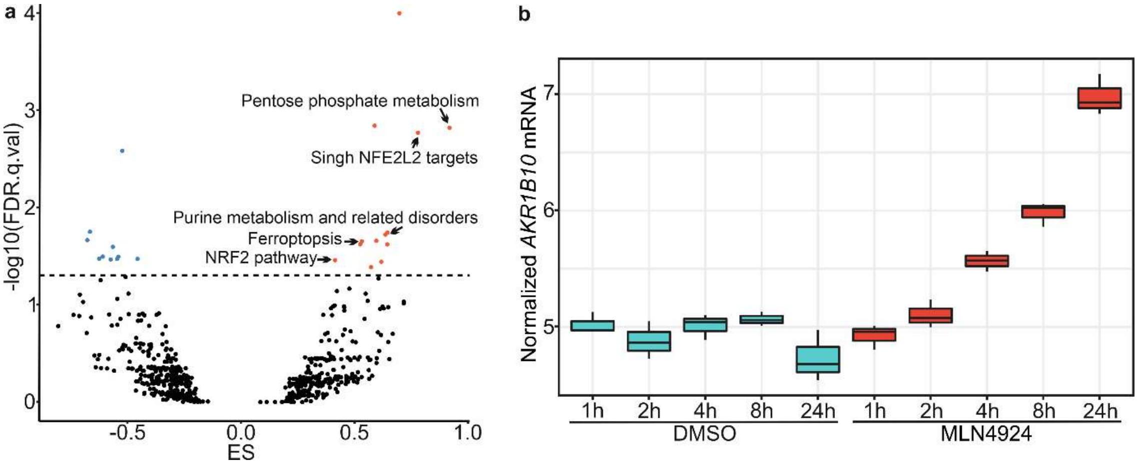

Supplementary Figure. 5. **Inhibition of NEDD8-activating enzyme leads to activation of NRF2 target genes in melanoma cells. a)** GSEA analysis revealed statistically significant upregulation of gene sets associated with the NRF2 pathway in melanoma cells treated with the NEDD8-activating enzyme inhibitor MLN4924. **b)** Expression of AKR1B10 mRNA increased when melanoma cells were treated with NEDD8-activating enzyme MLN4924.
